# Pyrene morphology and molecular identification of some garden ornamental palms of the family Arecaceae based on the plastid *rbcL* gene in Egypt morphological and molecular identification of ornamental palms (Arecaceae) in Egypt based on pyrene traits and *rbcL* gene sequence

**DOI:** 10.1186/s12870-025-07788-w

**Published:** 2025-12-19

**Authors:** Marwa M. El-Demerdash, Ashraf S. A. El-Sayed, Marwa A. Yassin, Heba H. Elsehely

**Affiliations:** 1 Botany and Microbiology Department, Faculty of Science, Zagazig University, Zagazig, 44519 Egypt; 2 Botany and Microbiology Department, Faculty of Science, Tanta University, Tanta, 31111 Egypt

**Keywords:** Palms, Arecaceae, Pyrene, Plastidal *rbcL* gene, PCA analysis, Phylogenetic analysis

## Abstract

The ornamental palms represent a diverse species in the national botanical gardens, and roadsides; however, the accurate identification of the palm trees (Arecaceae) is a problematic due to the numerous overlapped morphological traits, especially with the environmental conditions. So, the objective of this study was to implement the different morphological traits, especially based on the pyrene morphology, with the molecular barcoding markers of the plastid *rbcL* on delineation and revising the taxonomical identification of the most common Palm trees in Egypt in addition to their pharmacological and Ethnobotanical applications. An obvious variation on the surface of pyrenes among the studied Palm taxa ranged from ovoid to globose or discoid, with brown to pale brown, was recorded. The pyrene's fruit dimensions were ranged with *S. yaba* Becc. (5.65 × 6.85 mm), *Washingtonia robusta* (7.19 × 4.4 mm) and *Sabal palmetto* (Walter) Lodd. ex Schult. & Schult.f. (7.24 × 9.58 mm), while *Syagrus romanzoffiana* (Cham.) Glassman is (19.8 × 12.15 mm). The color of the pyrene of *W. robusta*, *S. romanzoffiana*, and *Livistona decora* (W.Bull) Dowe was brown, while was dark brown in *Butia capitata* (Mart.) Becc*. Sabal yapa* and *S. palmetto.* The SEM analysis of the pyrene surface microsculpture, the studied taxa of *S. palmetto, S. yaba, Livistona, Brahea,* and *Sabal* could be easily delimited at the generic level. The taxonomical identification of plant taxa based on their morphological characteristics, such as color, surface smoothness, and geometric shapes, was confirmed based on their molecular barcoding. The Principal Component Analysis (PCA) scatter plot based on the morphological traits distinguishes the taxa of tribe Cocoeae, subfamily Arecoideae and taxa of tribe Corypheae, subfamily Coryphoideae. From the UPGMA dendrogram based on the micromorphological characteristics, the studied taxa were grouped into two major clusters (I, II), the cluster I includes *S. palmatto, S, yaba* and *W. robusta* which belongs to subtribe Sabalinae, tribe corypheae, while cluster II includes *L. decora, L. chinensis,* (Jacq.) R.Br. ex Mart.and *B. armata* which belongs to subtribe Livistoninae and tribe Corypheae. Thus, the classification of the experimental plants based on the morphological traits of pyrene fruit microsculpturing was closely matched with the molecular barcoding based on the *rbcL* sequences.

## Introduction

The family Arecaceae Berchtold. & Presl (Palmae Juss.) comprises about 185 genera and 2600 species [1], mostly confined to the tropics of hemispheres and warm temperate regions [1, 2]. In Egypt, the ornamental palms represent a crucial constituent of the national botanical gardens, private gardens, roadsides and even the orchards, in addition to the implementation of landscaping projects [3]. The economic importance of the palm species has been confined to food, a source of oil, and supplying material for thatching, making hats, mats, and baskets [4, 5]. Practically, the palm plant and its derivatives have been used in the production of pulp, medicine, and colorants [6, 7]. However, the accurate identification of the palm trees (Arecaceae) at botanical gardens is partially problematic, as numerous botanical garden collections of the palm trees were partially misidentified and do not resolve to the species or genus level [8]. The misidentification challenges of this family could be due to the numerous morphological traits, a diverse number of recognized species of palm. The family Arecaceae comprises by five subfamilies [2], as follows: Arecoideae, Calamoideae, Ceroxyloideae, Coryphoideae, Nypoideae with only one genus *Nypa* Steck and Phytelephantoideae with three genera, namely *Ammandra* O.F.Cook, *Phytelephas Ruiz & Pav,* and *Aphandra* Barfod. The subfamily Phytelephantoideae has been subsequently excluded, while the other five subfamilies were included in the Palmae [9, 10]. The palm fruit is either a berry or drupe [4], the endocarp may be thick and stony in the drupaceous fruit to thin papery or fleshy in the berry fruit [11]. In the drupaceous fruit the stony endocarp is usually adhering to the seed to form a pyrene [2, 12]. The palm seeds are the valuable source for the propagation and conservation of germplasm [13]. So, the seeds/pyrenes morphology could have a reasonable contribution to the conservation and protection programs of the genetic resources [14–16]. Fruit morphology has been used as robust taxonomic tools in plant classification at the families, genera and species levels [17, 18]. Environmental factors have less impact on the seed characteristics, that frequently reflect the numerous genetic variations [19], so it is a crucial in identification and delineation of numerous species and genera of angiosperms [20–23]. Several reports describing the palm seeds' structure, histo-chemistry, germination, dormancy, storage of seeds, in addition to the seedling development, morphology, and anatomy [24–29]. Molecular markers have recently become widely utilized in diversity, conservation, phylogenetic, genetic and ecological, and taxonomical studies among different organisms [30–32]. Numerous cutting-edge studies have used DNA barcoding to validate plant identification, particularly for specimens in the early stages of growth, when many botanical garden collections lack identifying floral features. DNA barcoding has been used to determine species relationships within the palm family, especially with high species diversity and cultural significance [33, 34]. Among the common molecular barcodes, the plastid genes *rbcL* and *matK* were frequently used for discrimination between the closely related taxonomically species of Palm [8, 35]. Recently, the sequence of Start codon targeted polymorphisms “SCoT polymorphisms”, has been considered as a new robust and reproducible marker due to their dependence on the conserved area that flanks the ATG “start codon” in the plant genes [36–38]. Furthermore, SCoT polymorphism exhibits reduced levels of recombination between genes/traits in comparison to other markers [38]. Eight species represent the genera *Brahea*, *Butia*, *Livistona*, *Sabal* Adans *Syagrus* and *Washingtonia* were used as a case study. These palm species plant were recognized with pyrene formation; however, there is a scare studies describing the traditional taxonomical identification, especially in correlation with pyrene formation, in Egypt. So, the accurate identification of the different species of Palm trees, especially in Egypt, remains equivocal. So, the objective of this work was to implement the different micro and macro-morphological traits, seed microsculpturing, in addition to the molecular barcoding using plastid *rbcL* sequence in revising the identification and delineations of the selected Palm species in Egypt.

## Materials and methods

### Sample collection and geographical distribution

Eight palm species were collected from the Orman botanical garden, Giza, Egypt have been selected for this study (Table 1). The ripe, drupaceous fruits of the investigated palm species were picked from individuals of the investigated palm species during November 2016, the fruits were collected in plastic bags and kept in the refrigerator. Many of the collected fruits, from each species, were de-pulped by hand to obtain the pyrenes (seed-like bodies in which the seeds tightly connected and enclosed by the endocarp). Vouchers of the fruits and seed samples (i.e. pyrenes) were deposited at Herbarium of Botany Department, Faculty of Science, Zagazig University (HBD-ZU), Egypt.

**Table 1:** The studied species of the ornamental palms according the conspectus of classification of palms by Dransfield andUhl [2]

Subfamily	Tribe	Subtribe	Genus	Species	Vouchers numbers
Arecoideae	Cocoeae	Butiinae	*Butia* Becc	*Butia capitata* (Mart.) Becc	0001014CP0369
			*Syagrus* Mart	*Syagrus romanzoffiana* (Cham.) Glassman	001762SC00194
Coryphoideae	Corypheae	Livistoninae	*Brahea* Mart	*Brahea armata* S. Watson	000970CA0225
			*Livistona* R.Br	*Livistona chinensis (Jacq.) R. Br. ex Mart*	001299LC0120
				*Livistona decora (W. Bull) Dowe*	001301LD0122
			*Washingtonia* H. Wend	*Washingtonia robusta* H. Wendl	002078WC0004
		Sabalinae	*Sabal* Adans	*Sabal palmetto* (Walter) Lodd. ex Schult. & Schult. f. *Sabal yaba* C. Wright ex Becc	001520SP0079 001520SY0090

### Morphological and molecular identification of the samples

#### Microscopical analyses of pyrenes

The dimensions; length and breadth of the pyrenes, were measured as an average of 10 specimens for each species using the vernier caliper, for the dimension measurement. The macromorphological characters of the pyrenes, such as shape, colour, surface structures, operculum, and germ pores (orifices) were examined by the stereoscopic light microscope (GX, XTL-101, Suffolk, UK), were photographed using digital camera. The pyrenes for each species were mounted on stubs, then coating with gold for JEOL-JFC 1100 E sputtering. The pyrenes were examined using a Scanning Electron Microscope (JEOL-JSM-5300) with an accelerating voltage of 20 kV at the Faculty of Science, Alexandria University, Alexandria, Egypt.

The microsculpturing details of pyrenes fruit, showing their structural features were illustrated. The terminology/parameters for describing the observed sculpturing patterns were addressed [39, 40]. The PAST software package (Version 4.3c) was used for description of the macro- and micromorphological traits as recorded in Tables 2, 3) [41]. Data matrix was investigated by a multistate matrix, followed by clustering analysis with UPGMA (Unweighted pair group method with arithmetic mean).

**Table 2:** List of primer Scot and *rbcL*

Primer name	Primer sequence (5'−3')
Scot −01	CAACAATGGCTACCACCA
Scot-02	CAACAATGGCTACCACCC
Scot-03	CAATGGCTACCACTAGCG
Scot-04	ACAATGGCTACCACTAGG
Scot-52	ACAATGGCTACCACTGCA
rbcLF	ATGTCACCACAAACAGAGACTAAAGC
rbcLR	TCGCATGTACCTGCAGTAGC

**Table 3:** Pyrene morphological features of the studied palm species

Species	Shape	Colour	Size mm			External surface structures under LM				
			Length	Width	Grade*	Groove	Operculum		Germ pore	
							Shape	Site	Number	Site
*Brahea armata*	Discoid-subglobose	Pale brown	15.35	15.93	Medium	Absent	Absent	Absent	Absent	Absent
*Butia capitata*	Ovoid-globose	Dark brown	15.36	12.66	Medium	Absent	Absent	Absent	3	Sub-basal
*Livistona chinensis*	Ovoid	Pale brown	15.71	10.33	Medium	Absent	Absent	Absent	1	Sub-basal
*Livistona decora*	Globose	Brown	10.24	11.28	Medium	Absent	Absent	Absent	1	Sub-basal
*Sabal palmetto*	Discoid-subglobose	Dark brown	7.24	9.58	Small	Absent	Dome shaped	Lateral	Absent	Absent
*Sabal yaba*	Discoid	Dark brown	5.65	6.85	Small	Absent	Dome shaped	Lateral	Absent	Absent
*Syagrus romanzoffiana*	Ovoid-globose	Brown	19.88	12.15	Large	Absent	Absent	Absent	3	Sub-basal
*Washingtonia robusta*	Ovoid-globose	Brown	7.19	4.4	small	Present	Absent	Absent	Absent	Absent

^*^Concerning pyrene size, the following three pyrene size grades were considered: small-sized i.e. less than 8 mm long, medium-sized 8–16 mm long and large-sized pyrenes more than 16 mm long

### Molecular identification of the plants

The *rbcL* sequence was used to confirm the morphologically identified plant samples, based on the genomic plant DNA as a PCR template [42]. The CTAB reagent was used for the plant genomic DNA extraction [43], with minor modifications. Briefly, 500 μl of the CTAB solution was added to 0.1 g of the liquid nitrogen-homogenized plant tissues, with gentle mixing, followed by centrifugation at 10,000 rpm for 10 min. The supernatant was mixed with 1/1 v/v chloroform, centrifuged at 10,000 rpm for 10 min, the upper layer was mixed with absolute ethanol (1/1 v/v), left at −20 °C for 12 h, and the DNA was pelleted by centrifugation for 10 min at 10,000 rpm, and washed with 70% ethanol. The DNA pellets were dissolved in 50 μl distilled water and stored at −20 °C. The DNA purity was assessed by 1.5% agarose gel, compared to 1 kb DNA ladder (Cat.# PG010-55DI). The *rbcL* and SCoT primers were mentioned in Table 2. Five Start Codon Targeted (SCoT) primers were used frequently to assess the genetic fidelity of the plants [44]. The PCR reaction consists of i-Taq™ (Cat. # 25,027), gDNA, primer (10 pmol), and distilled water in 20 μl reaction volume [37]. The PCR conditions were set up as 94 °C for 2 min, 35 cycles of denaturation 94 °C for 20 s, annealing at 51 °C for 30 s, extension at 72 °C for 1 min, and final extension at 72 °C for 5 min. The PCR products were examined by agarose gel (1.5%) in 1 × TBE buffer (Cat.#AM9864), the amplicons were purified, and sequenced. The MEGA 6.0 software program was used to match the recovered sequences with the Clustal W muscle method [45] with 1000 bootstrap replications, with the Maximum Likelihood technique to construct the phylogenetic relatedness [46]. The *rbcL* sequences were refined using BioEdit version 7.2.5 [47], and analyzed by MEGA X software [48]. The pairwise sequence divergence across the examined taxa in the *rbcL* regions was computed by the Maximum Composite Likelihood (MCL) compared with the sequences from other species by the Basic Local Alignment Search Tool. The transition/transversion ratio ti/tv was estimated by the following formula R = [A*G*k1 + T*C*k2]/[(A + G)*(T + C)] with A,G,C,T as the corresponding frequencies of the four nucleotides [49]. The sequences were analyzed with DnaSP software version 4.0 [49] to estimate the polymorphism indices. The average of nucleotide differences (k) and the minimum number of recombination events (Rm) were estimated. Selection neutrality was tested by both Tajima’s D [50] and Fu and Li’s D* and F* methods [51]. Endmemo software was used to estimate the GC contents and amplicon size (http://www.endmemo.com/bio/gc.php) (Table 4).

**Table 4:** Pyrene microsculpturing details of the studied palm species

Species	Surface pattern	Anticlinal walls	Inner periclinal walls	Wax deposits	
*Brahea armata*	Irregularly reticulate with collapsed cells in some parts	Thick, Raised, straight-ill defined (collapsed)	Concave, granulate-microstriate	Sparse granules variable in both shape and size	Figure 2A
*Butia capitata*	Commonly featureless, but sometimes with broken (collapsed) elongate cells	Commonly broken (collapsed), but sometimes arising Thick, Raised, straight	Concave, irregular	Sparse minute granules	Figure 2B
*Livistona chinensis*	Alveolate (i.e. honey-combed)	Thick, Raised, Straight-slightly undulate	Concave, micro-perforate(distinctive character)	Variably-sized rods, rectangular blocks and large lumps of aggregated granules filling the lumen of many cells	Figure 2. C
*Livistona decora*	Commonly featureless but showing with rugosity	Undulate	undulate	Sparse granules variable in both shape and size	Figure 2D
*Sabal palmetto*	Reticulate (with thick, raised, straight anticlinals and the inner periclinal walls concave and with (compactly dense granulate	Thick, raised, straight	Concave, compactly dense granulate	Sparse granules variable in both shape and size	Figure 2E
*Sabal yaba*	Reticulate-foveolate but commonly the pattern occluded in most parts by heavy depositions of cuticle	Thick raised, straight (in some parts)	Shallow concave, smooth to minutely granulate (some parts where the cells not occluded by cuticle)	Sparse minute granules	Figure 2F
*Syagrus romanzoffiana*	Collapsed reticulate-rugose	Thick, raised, straight	Shallow raised, frequently perforate	Sparse granules variable in shape and size	Figure 2G
*Washingtonia robusta*	Reticulate	Exaggeratedly thick, raised, straight, frequently incomplete (arrowed), dense transversely striated	Concave, Dense transversely striated	Sparse granules variable in shape and size	Figure 2H

### Clustering analysis

The PCR products of the SCoT “only the clear and reproducible bands” were scored as 0 or 1, to construct the samples' binary matrix. The clustering analysis was conducted using PASW Statistics version 18.0 [52], and phylogenetic similarity was generated using the UPGMA method, based on PCA using Euclidean analyses [53].

## Results and discussion

### Macro and micromorphological features of pyrene of the studied palm species

**Fig. 1:**
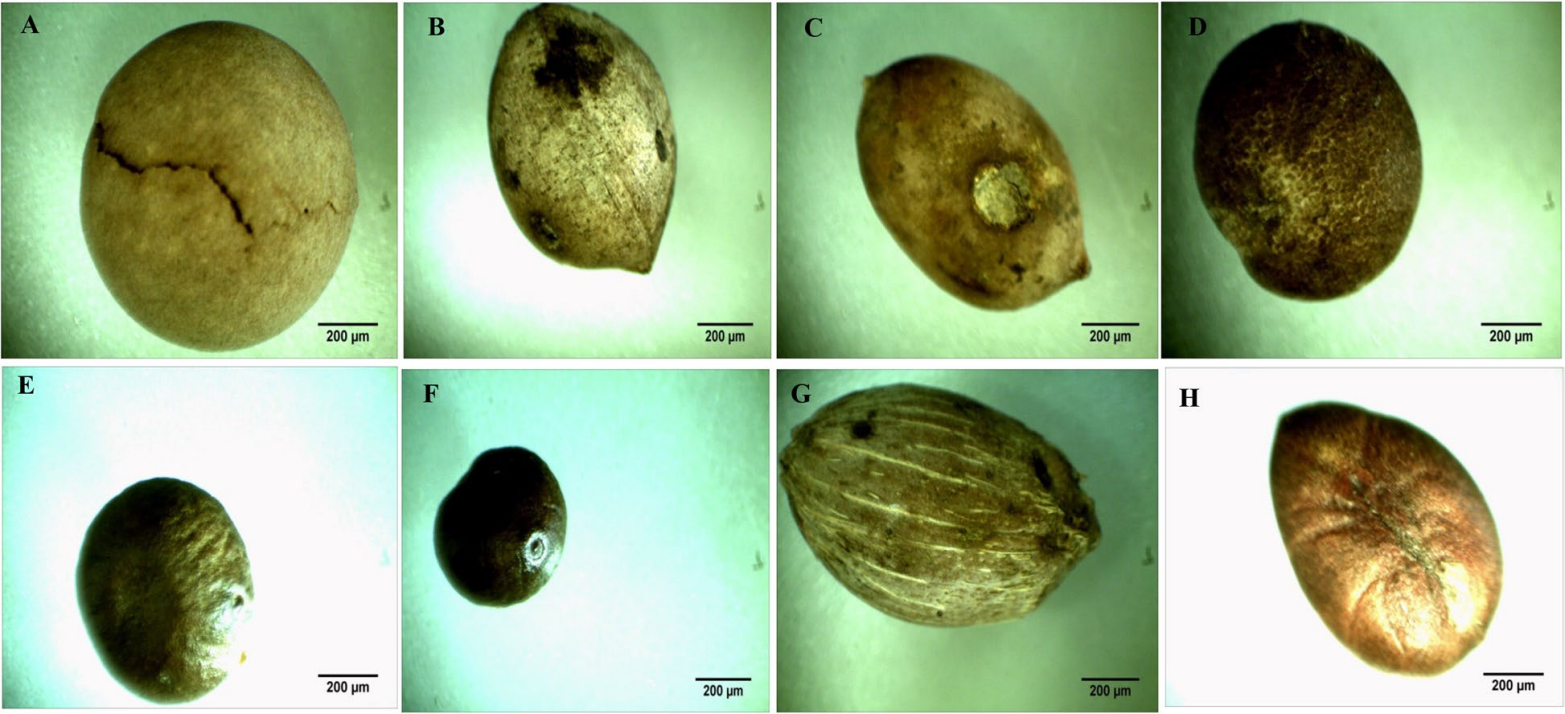
Pyrene macrosculpturing details of the studied palm species.**A** *Brahea armata*, **B** *Butia capitata*, **C** *Livistona chinensis*, **D** *Livistona decora*,**E** *Sabal palmetto*, **F** *Sabal yaba*, **G** G1-*Syagrus romanzoffiana*, **H** *Washingtonia robusta*

**Fig. 2:**
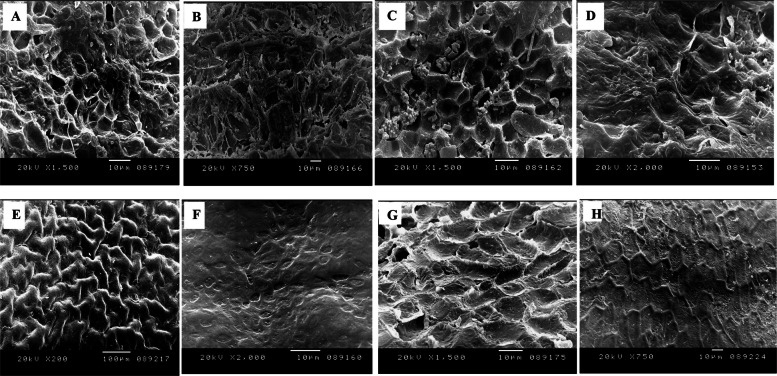
Pyrene microsculpturing details of the studied palm species.**A** *Brahea armata*, **B** *Butia capitata*,**C** *Livistona chinensis*, **D** *Livistona decora*, **E** *Sabal palmetto*, **F** *Sabal yaba*, **G** *Syagrus romanzoffiana*, **H** *Washingtonia robusta*

The morphological traits of the pyrene fruit of the selected Palm species were recorded. Since, the fruit characteristics provide a more reliable criteria for the plant identification and subsequent classification other than the morphological traits (plant height, number of leaves, trunk thickness, petiole length and number of leaflets) [54–56]. Additionally, the plant morphological characteristics depends on the plant age, and consequently they need to be inspected repeatedly [57], since these traits are usually controlled by the gene expression that affects the climatic and ecological conditions [58, 59]. Seed morphometric serves as a valuable tool for the species identification of palm diversity such as the Amazon basin. [60]. The endosperm cell wall of the palm seeds is often thick for the deposition of storage polysaccharides, mostly the hemicelluloses mannan [61]. From literature, few studies on the Palm taxonomical classification based on the pyrene morphological features and molecular barcoding were reported in Egypt [62]. From the microscopical features of pyrene fruits of the selected plant taxa by the stereomicroscopy and SEM, various valuable characters were explored for identification and separation of these taxa. The salient morphological features of the pyrenes of the studied species include the shape, colour, size, surface structures; groove, operculum and germ pores as observed by the stereomicroscope (Tables 2, 3). The micromorphological traits and microsculpturing details of the pyrene by the stereomicroscope was observed (Fig. 1). There is an obvious variation on the surface of pyrenes among the studied Palm taxa ranged from ovoid to globose or discoid, and with some intergraded forms, the pyrene colour was brown, to pale brown or dark brown. The seeds of Arecaceae are usually circular, elliptic, globose, irregularly globose, ovoid, broadly ovoid, piriform, or rounded [9, 60]. The length of the pyrenes seems to be a good taxonomic tool in differentiating the genus level as ranged from 5.65 mm to 19.88 mm. The dimensions of the pyrene's fruits were different among the tested Palm taxa, *Sabal yaba* (5.65 × 6.85 mm), *Washingtonia robusta* (7.19 × 4.4 mm), *S. palmetto* (7.24 × 9.58 mm), while for *Syagrus romanzoffiana* was (19.88 × 12.15 mm), *Brahea armata* (15.35 × 15.93 mm), *Butia capitata* (15.36 × 12.66 mm), *Livistona chinensis (*15.71 × 10.33 mm), and *L. decora (*10.24 × 11.28 mm). Fruiting traits such as fruit diameter, length, and size revealed clear differences between date palms [62]. The color of the pyrene of *W. robusta*,* S. romanzoffiana*, and *L. decora* was brown, while was dark brown in *B. capitata*, *S. yapa,* and *S. palmetto.* During germination, the seeds of palm often possess a thick seed coat with a single operculum, corresponding to the scar adjacent to the micropyle [63–65]. Also, three orifices or germ pores were observed at the sub-basal on the pyrenes of *B. capitata* and *S. romanzoffiana* (Fig. 1B,G). However, in *Livistona chinensis* and *L. decora,* only one sub-basal germ pore is present (Fig. 1C,D). The microsculpturing details of the pyrene surface of the tested palm species were visualized by the SEM (Fig. 2), showing the four sculpturing patterns concomitantly with integrated patterns that were designated as follows: Type 1, Alveolate (i.e. honey-combed) in *L. chinensis* anticlinal walls, thick, raised, straight-slightly undulate inner periclinal walls, concave, micro-perforate, wax deposits variably-sized rods, rectangular blocks and large lumps of aggregated granules filling the lumen of cells (Fig. 2C). Type 2, Reticulate pyrene in *Brahea armata* with irregularly reticulate collapsed cells, thick, raised, straight-ill defined (collapsed) anticlinal wall thick, raised, straight-ill defined (collapsed), inner periclinal walls, concave, granulate-microstriate (Fig. 2A). While, the pyrene of *S. palmetto* characterized with wax deposits sparse granules variable in both shape and size reticulate (with thick, raised, straight anticlinals and the inner periclinal walls concave and with compactly dense granulate (Fig. 2A, E). In *W. robusta,* the pyrene was characterized with anticlinal wall exaggeratedly thick, raised, straight, dense transversely striated inner periclinal walls concave, dense transversely striated wax deposits sparse granules variable in shape and size (Fig. 2H). Type 3 in *Sabal yaba,* the pyrene surface was reticulate-foveolate with heavy depositions of cuticle anticlinal walls, thick, shallow raised, straight, inner periclinal walls shallow concave, smooth to minutely granulate partially occluded with cuticle, wax deposits sparse minute granules (Fig. 2F). Type 4, reticulate-rugose pyrene surface in *Syagrus romanzoffiana* anticlinal walls thick, raised, straight inner periclinal walls shallow raised, frequently perforate wax deposits sparse granules variable in shape and size (Fig. 2H). Details of the Pyrene microsculpture under SEM have been found very useful in identifying many of the examined pyrene of palm plants, in the separation of their corresponding species. In addition, the two studied taxa of* S. palmetto* and *S. yaba,* species of *Livistona, Brahea,* and *Sabal* could be easily delimited at the generic level by their pyrenes' surface microsculpture. Similar investigations have used pyrene surface microsculpture to delineate 17 species of *Rubus* sp L cultivars based on raphe form (straight, concave, or convex) [64]. Cultivars within each group can be distinguished by seed morphology, dimensions, color, and seed-coat texture [65, 66]. Exploring the seeds shape is an interesting characteristic to distinguish the members of Arecaceae at the species level. The molecular barcoding in correlation with the identity of the geometric shapes were used to validate the morphological characteristics such as color, surface smoothness and striation [59]. The UPGMA dendrogram conducted from the macro- and micromorphological characteristics were shown in Fig. 1, categorized all the examined taxa into two major clusters (I,II). Cluster I includes *S. palmatto, S, yaba* and *W. robusta* that belong to tribe corypheae. Cluster II includes (subcluster 1) *L. decora, L. chinensis,* and *Brahea armata,* which belong to subtribe Livistoninae, tribe Corypheae, subfamily Coryphoideae. Subcluster 2 includes *B. capitata* and *S. romanzoffiana.*

### PCA analysis

The PCA analysis represents the distribution and occurrence of various Macro- and micromorphological features of the studied species. The PCA scatter plot clearly distinguishes between taxa of tribe Cocoeae, subfamily Arecoideae and taxa of tribe Corypheae, subfamily Coryphoideae, indicating the features. The species of *B. capitata* and *S. romanzoffiana* were grouped together; the species of* L. decora, L. chinensisa, B. armata, S. palmatto, S. yaba* and *W. robusta* was separated into a distinct group (Fig. 1). The interspecific genetic divergence refers to the genetic variation within the species, with the clear separation of the two tribes as coincident with the criteria of Macro-micromorphological features. The separation of tribe Cocoeae and tribe Corypheae into two groups prove the segregation of the tribes according to Dransfield and Uhl [2].

### Molecular barcoding of the experimental plants species using the chloroplast gene rbcL

**Fig. 3:**
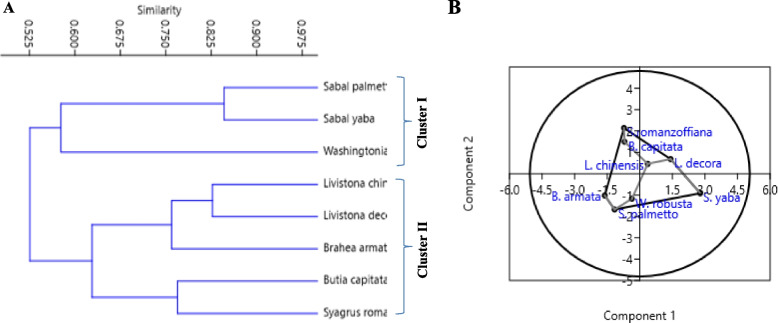
**A** UPGMA analysis of the experimented PALM species, based on their pyrene Macro-micromorphology characters. **B** PCA analysis of the studied taxa

The identity of the experimented plants was verified based on the chloroplastic *rblcL,* comparatively to their morphological traits. The traditional taxonomical features that are based on the morphological traits have been accepted in traditional taxonomy, with efficacy; however, the major challenges of this approach are the variations of these features with the environmental conditions and stages of the plant growth [67, 68]. So, the recent tools of molecular identification of the plants based on the sequences of some conserved genes and protein coding genes have been used as an authenticating tools for the traditional taxonomy [62, 69, 70]. The morphological identity of the studied plant taxa *B. armata, B. capitata, L. chinensis, L. decora, Sabal palmetto, S. yaba, S. romanzoffiana* and *W. robusta* were molecularly confirmed based on the sequence of their *rbcL* domain. The PCRamplicons of the *rbcL* of the experimental plants were separated with approximately 700 bp (Fig. 3A), and the sequences were non-redundantly BLAST searched on the NCBI. The *rbcL* sequences of *B. armata, B. capitata, L. chinensis*, *L. decora, Sabal palmetto, S. yaba, S. romanzoffiana* and *W. robusta* were deposited to the Genbank with accession numbers PP598864, PP496909, PP598865, PQ306609, PQ252347, PP716098, PP496910 and PP716097, respectively. The sequence of the chloroplastic gene *rbcL* was authenticated frequently a relevant tool for identification of plants confirming the traditional taxonomical traits [62, 71, 72]. DNAbarcoding is confirmatory tool to morphological identification for the different plant species [69, 70]. The sequence of the chloroplastic *rbcL* has been used as a plant DNAbarcoding due to its universality across the diverse plant genera and its capacity to distinguish closely related species [73–75]. The obtained sequences demonstrating the differences in the GC content of the experimental plants were summarized in Table 5. The sizes of *rbcL* sequences were varied from 626 to 679 bp in *Livistona decora* MM and *Butia capitata* MM1, respectively (Table 5), The nucleotide frequencies are 27.84% for adenine (A), 28.34% for thymine/uracil (T/U), 21.11% for cytosine (C), and 22.71% for guanine (G). The ratios of transition to transversion rates were k1 = 0.974 for purines and k2 = 2.612 for the pyrimidines (Table 6). The overall transition/transversion bias is R = 0.871, calculated as R = [A*G*k1 T*C*k2]/[(A + G) *(T + C)]. This analysis involved 8 nucleotide sequences. Codon positions included were 1 st + 2nd + 3rd + Noncoding (Table 5). The Transversion rates of *Phoenix* species were 4.39 for T to G and C to G, 5.51 for A to T and G to T, 5.67 for T to A and C to A, and 4.05. The transition rates were as follows: 14.54 from A to G, 26.74 from C to T, 19.61 from T to C, and 18.72 from A to G. The frequencies of nucleotide substitution were T/U = 28.10%, C = 20.36%, G = 22.66%, and A = 28.88% [35]. The study included 35 nucleotide sequences of the date palm grown in Siwa Oasis has an estimated Transition/Transversion bias (R) of 0.74, with nucleotide frequencies of 28.21% for adenine (A), 28.58% for thymine/uracil (T/U), 20.95% for cytosine (C), and 22.26% for guanine (G) [62]. Our findings support the idea that a useful barcode needs to have higher interspecific divergence than intraspecific divergence in order to differentiate between species [76]. Evolutionary analyses by MEGA6 showed that there were 667 conserved sites, 13 variables sites, and 3 singleton loci in the *rbcL* nucleotide alignment's fluctuating composition into the 680-character matrix.

**Table 5:** Length and GC contents of the *rbcL* sequences of the palm species

Scientific name	**NCBI Accession**	**Length bp**	**GC** %
1- *Butia capitata* (Mart.) Becc	PP496909	679 bp	44.03
2- *Syagrus romanzoffiana*	PP496910	664 bp	44.27
3- *Brahea armata*	PP598864	678 bp	42.92
4- *Livistona chinensis*	PP598865	679 bp	43.29
5- *Livistona decora* (W. Bull) Dowe	PQ306609	626 bp	43.13
*6- Sabal palmetto* (Walter)	PQ252347	664 bp	43.67
7- *Sabal yaba*	PP716098	653 bp	44.25
8- *Washingtonia robusta* H. Wendl	PP716097	660 bp	43.63

**Table 6:** Maximum composite likelihood estimate of the pattern of nucleotide substitution of *rbcl* gene of studied taxa

	A	T	C	G
A	-	*7.49*	*5.58*	**5.84**
T	*7.36*	-	**14.57**	*6*
C	*7.36*	**19.56**	-	*6*
G	**7.17**	*7.49*	*5.58*	-

Each entry shows the probability of substitution (r) from one base (row) to another base (column) [1]. For simplicity, the sum of r values is made equal to 100. Rates of different transitional substitutions are shown in **bold** and those of transversionsal substitutions are shown in *italics*

**Fig. 4:**
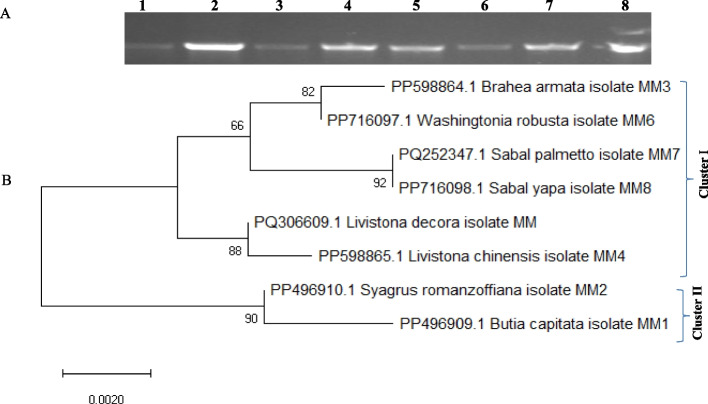
PCR amplified products using *rbcL* specific primers. M representing DNA ladder, lane 1–8 representing PCR amplification. 1- *Butia capitata*, 2- *Syagrus romanzoffiana,3-Brahea armata*, 4-*Livistona chinensis*, 5-*Livistona decora*, 7- *Sabal palmetto*,8- *Sabal yaba*, 9-*Washingtonia robusta*

**Fig. 5:**
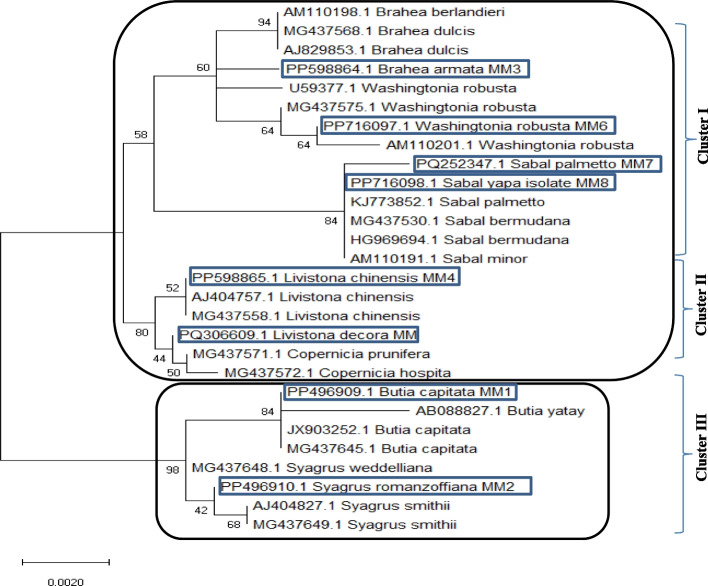
Neighbor-joining relatedness of the eight studied species based on the *rbcl* sequence with the Genbank deposited *rbcL* sequences of the different species

From phylogenetic analysis, the tested palm taxa were separated into two different clusters (I and II) of the subfamily Arecoideae and Coryphoideae (Fig. 4). The cluster II include *Butia capitata* MM1, and *Syagrus romanzoffiana* MM2, that belongs to the tribe Cocoeae and subtribe Butiinae. The cluster I includes the two sub-clusters 1 and 2. The sub-cluster 1 has *Livistona decora* MM PQ306609 and *L. chinensis* MM4 PP598865 that belongs to the subfamily Coryphoideae, tribe Corypheae subtribe Livistoninae. The sub-cluster 2 contains *Sabal yapa* MM8 PP716098, *Sabal palmetto* MM7, *Washingtonia robusta* MM6 PP716097.1, and *Brahea armata* MM3 PP598864, that belongs to the tribe Corypheae and sub-tribe, subfamily Coryphoideae. So, from the phylogenetic analysis, the molecular identification of the plant being matched with those reported from the morphological classification. According to the Maximum likehood (ML) analysis, which completely congruent with the allocation of these taxa into their corresponding subfamily as well as tribe ensure the effectiveness of the *rbcL* barcode in identifying, and classifying the genetic relationships of palm species [2]. Similar results, ensuring the molecular barcodes of Palm based on the *rbcL* genes were reported for verification of the traditional taxonomical identification of plant [8, 35, 62, 77]. The phylogenetic analysis of the tested Palm taxa based on the *rbcL* sequences were conducted, three clusters were appeared (I, II, III) (Fig. 5). From the phylogenetic analysis, the *rbcL* sequence of *B. armata* PP598864.1 had a significant similarity (65%) with *B. dulcis* AJ829853.1, *B. dulcis* MG437568.1, *B. berlandieri* AM110198.1, *W. robusta* U59377.1, MG437575.1, AM110201.1, with E-value zero. The similarity of *S. palmetto* PQ252347.1 MM7 and *S. yapa* PP716098.1MM8 was 84% with *S. palmetto* KJ773852.1, *S. bermudana* MG437530.1, *S. bermudana* HG969694.1 and *S. minor* AM110191.1. *Livistona chinensis* PP598865.1MM4 and *L. decora* PQ306609.1 belongs to the Cluster II with similarity 52% with *L. chinensis* AJ404757.1, MG437558.1, *Copernicia prunifera* MG437571.1 and *C. hospita* MG437572.1. From the phylogenetic analysis based on *rbcL* sequence, *B. capitata* PP496909.1 had 84% similarity with *B. yatay* AB088827.1, *B. capitata* JX903252.1 and MG437645.1, while *S. romanzoffiana* PP496910.1MM2 had 42% similarity with *Syagrus smithii* AJ404827.1, and MG437649.1, that belongs to the cluster III. The selective neutrality assay for the *rbcL* sequences is one of the most successful approaches for exploring the evolutionary domains on specific genes for plant taxonomy [78, 79]. To the best of knowledge, this is the first report addressing the evolutionary traits of Palm based on the *rbcL* sequences comparing to the morphological traits, involving the neutrality assays. Selective neutrality for the detected variations was tested by both Tajima [50] methods to examine the null hypothesis. Tajima D is −0.013911 (Table 7). Fu and Li's D* [51] test statistic: -*: 0.89128. Statistical significance: *P* > 0.10 Fu and Li's F* test statistic: 0.83437 Statistical significance: Not significant, *P* > 0.10. The average number of pairwise nucleotide differences, k: 4.750, Nucleotide diversity, Pi: 0.007353, Number of segregating sites is 0.019118.Theta estimated from k: 4.750. Theta estimated from Eta(s): 1.750.Theta estimated from Eta: 4.242.

**Table 7:** Results from Tajima's neutrality test of *rbcl* sequence

*m*	*S*	*p*_s_	*Θ*	*π*	*D*
8	13	0.019118	0.007373	0.007353	−0.013911

### Scot analysis of the tested palm plants

**Fig. 6:**
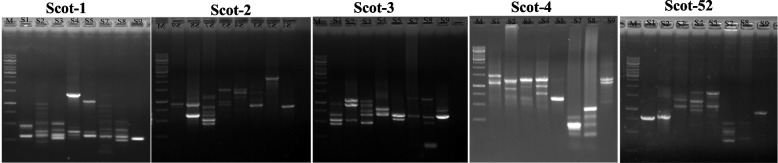
SCoT analysis of the experimented plants using the genomic DNA as a PCR template isolated from the fresh leaves using the primers Scot-1,2,3,4,52 primer. M: marker 1 kb DNA ladder. The eight plant samples *Butia capitata* (1), *Syagrus romanzoffiana* (2)*, Brahea armata* (3), *Livistona chinensis* (4), *Livistona decora* (5), *Sabal palmetto* (7), *Sabal yaba* (8), *Washingtonia robusta* (9)

**Fig. 7:**
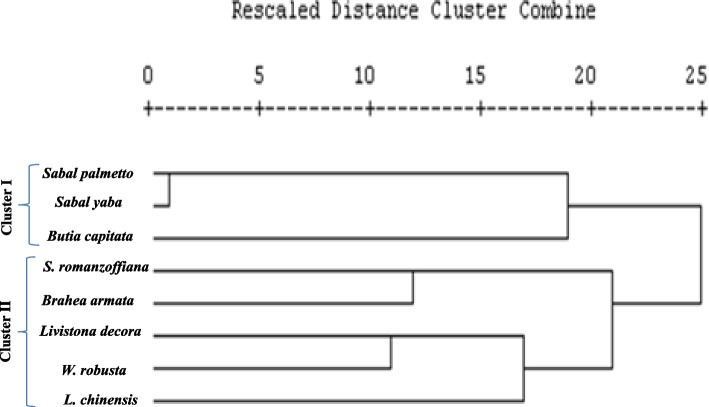
Dendrogram for the 8 Palm species constructed from the Scot data using Unweighed Pair-group Arithmetic average (UPGMA) and similarity matrices computed according to Dice coefficient

A SCoT approach has been recently used as recent molecular markers for confirming the traditional taxonomical traits of plants, assessing the genetic diversity among various plant species [80–85]. Five randomly selected primers were used for the Scot analysis (Tables 2), giving a reproducible profile in the species under investigation. The PCR amplicons of the SCoT corresponding to each primer for the experimental plants were illustrated in Fig. 6. A total of 60 bands were detected, comprising 11.8 polymorphic bands as amplicons from the PCR of genomic DNA across all species. Scot profile produced 16 distinct, reproducible bands, of which 15 were polymorphic and only one was monomorphic as generated by the SCoT-1 primer. The polymorphic percentage was 98.3%, the lowest ratio of polymorphism 93.75% was recorded by SCoT-01 (Table 8). The polymorphism ratio was 100% by SCoT-02, SCoT-03, SCoT-04 and SCoT-52 for the tested plants. The polymorphic information contents (PIC) was ranged from 0.33 to 0.45, with an average of 0.37, with the highest SCoT marker index (MI) (7.172) for the primer ABI-09 and the lowest (2.31) for the primer ABI-08. According to the Dice coefficient, the similarity index ranged between 0.103 and 0.706, as shown in Table 9. The highest similarity was 0.706 as reported between genotypes *S. palmetto* and *S. yapa,* while the lowest similarity 0.103 was observed between genotypes *Livistona decora* and *Sabal yaba* (Table 9). The phylogenetic analysis of SCoT was conducted utilizing UPGMA, with hierarchical clustering performed by SPSS 4.3e as shown in Fig. 7, giving two clusters (I, II). The SCoT analysis of the current genera was consistent with the morphological and conventional taxonomical characteristics and classification of the examined taxa. The cluster I has *S. palmetto*, *S. yapa* (belongs to Sabalinae subfamily Coryphoideae) and *Butia capitata* (belong to Cocoeae, sub family Arecoideae)*.* However, some discrepancies in the assignment of the taxa into their corresponding tribe were observed. The second subcluster (II) contains *S. romanzoffiana, Brahea armata, L. decora*, *W. robusta* and *L. chinensis.* Based on the traditional taxonomical traits, *Butia capitata* and *Syagrus romanzoffiana* were belongs to tribe Cocoeae [2] that being confirmed from the *rbcL* sequence, however, based on the SCoT analysis, *Butia capitata* was delineated away from this classification. This slight variation of the SCoT analysis and *rbcL* sequence could be ascribed to the technical dependency of SCoT analysis on single primer, unlike to the accuracy of the *rbcL* sequence that based on two primers, as a standard PCR reaction. So, from the morphological traits of pyrene and sequencing of the *rbcL,* the experimented plants species were categorized according to morphological traits [2].

**Table 8:** Primer code, the total number, monomorphic, polymorphic amplicons and the percentage of polymorphism as revealed by the SCoT analysis

Primers	Total no. of amplicons	Monomorphic amplicons	Polymorphic amplicons	% of polymorphism
Scot-01	16	1	15	93.75%
Scot-02	14	0	14	100%
Scot-03	10	0	10	100%
Scot-04	12	0	12	100%
Scot-52	8	0	8	100%
Average	12	0.2	11.8	98.33%

**Table 9:** Genetic similarity matrix among the studied taxa according to Dice coefficient from Scot

Proximity matrix								
Taxa	Matrix File Input							
	*Brahea armata*	*Syagrus romanzoffiana*	*Brahea armata*	*Livistona chinensis*	*Livistona decora*	*Sabal palmetto*	*Sabal yaba*	*Washingtonia robusta*
*Brahea armata*	1.000							
*Syagrus romanzoffiana*	.286	1.000						
*Brahea armata*	.471	.512	1.000					
*Livistona chinensis*	.333	.256	0.421	1.000				
*Livistona decora*	.400	.353	0.364	.414	1.000			
*Sabal palmetto*	.300	.327	0.250	.318	.103	1.000		
*Sabal yaba*	.486	.348	.356	.244	.222	.706	1.000	
*Washingtonia robusta*	.333	.242	.500	.429	.522	.105	.171	1.000

## Conclusion

The ornamental palms represent a crucial constituent of the national botanical gardens, in addition to the landscaping projects, in Egypt. However, the accurate identification of the palm trees is partially problematic at the species level due to the diversity of the various morphological traits especially the environmental conditions. The morphological features and micro-sculpture surface of the Palm pyrene have been used as recent taxonomical tools for Palm identification to the species level. The taxonomical traits of the experimental plants *Brahea*, *Butia*, *Livistona*, *Sabal*, *Syagrus* and *Washingtonia* were completely confirmed from the molecular barcoding based on the *rbcL.* To the best knowledge, this is the first study implanting the traditional taxonomical traits with the molecular barcoding of *rbcL* sequence to revise the identification of the Palm plants to the species level.

## Data Availability

All datasets generated for this study are included in the article. Butia capitata isolate MM1 ribulose-1,5-bisphosphate carboxylase/oxygenase large subunit (rbcL) gene, partial cds; chloroplast GenBank: PP496909.1 https://www.ncbi.nlm.nih.gov/nuccore/PP496909 Syagrus romanzoffiana isolate MM2 ribulose-1,5-bisphosphate carboxylase/oxygenase large subunit (rbcL) gene, partial cds; chloroplast GenBank: PP496910.1 https://www.ncbi.nlm.nih.gov/nuccore/PP496910 Brahea armata isolate MM3 ribulose-1,5-bisphosphate carboxylase/oxygenase large subunit (rbcL) gene, partial cds; chloroplast GenBank: PP598864.1 https://www.ncbi.nlm.nih.gov/nuccore/PP598864 Livistona chinensis isolate MM4 ribulose-1,5-bisphosphate carboxylase/oxygenase large subunit (rbcL) gene, partial cds; chloroplast https://www.ncbi.nlm.nih.gov/nuccore/PP598865.1/ Sabal yapa isolate MM8 ribulose 1,5-bisphosphate carboxylase-oxygenase large subunit (rbcL) gene, partial cds; chloroplast GenBank: PP716098.1 https://www.ncbi.nlm.nih.gov/nuccore/PP716098 Sabal palmetto isolate MM7 ribulose 1,5-bisphosphate carboxylase-oxygenase large subunit (rbcL) gene, partial cds; chloroplast GenBank: PQ252347.1 https://www.ncbi.nlm.nih.gov/nuccore/PQ252347 Washingtonia robusta isolate MM6 ribulose 1,5-bisphosphate carboxylase-oxygenase large subunit (rbcL) gene, partial cds; chloroplast GenBank: PP716097.1 https://www.ncbi.nlm.nih.gov/nuccore/PP716097 Livistona decora isolate MM ribulose-1,5-bisphosphate carboxylase-oxygenase large subunit (rbcL) gene, partial cds; chloroplast GenBank: PQ306609.1 https://www.ncbi.nlm.nih.gov/nuccore/PQ306609.
